# Management of a patient with multiple recurrences of fibromatosis (desmoid tumor) of the breast involving the chest wall musculature

**DOI:** 10.1186/1477-7819-4-32

**Published:** 2006-06-12

**Authors:** Stephen P Povoski, William L Marsh, Dimitrios G Spigos, Abbas E Abbas, Brentley A Buchele

**Affiliations:** 1 Division of Surgical Oncology, Department of Surgery, The Arthur G. James Cancer Hospital and Richard J. Solove Research Institute of The Ohio State University, Columbus, Ohio, 43210, USA; 2 Department of Pathology, The Arthur G. James Cancer Hospital and Richard J. Solove Research Institute of The Ohio State University, Columbus, Ohio, 43210, USA; 3 Department of Radiology, The Arthur G. James Cancer Hospital and Richard J. Solove Research Institute of The Ohio State University, Columbus, Ohio, 43210, USA; 4 Division of Thoracic Oncology, Department of Surgery, The Arthur G. James Cancer Hospital and Richard J. Solove Research Institute of The Ohio State University, Columbus, Ohio, 43210, USA; 5 Division of Plastic Surgery, Department of Surgery, The Arthur G. James Cancer Hospital and Richard J. Solove Research Institute of The Ohio State University, Columbus, Ohio, 43210, USA

## Abstract

**Background:**

Fibromatosis or desmoid tumor is a rare soft tissue tumor that lacks a metastatic potential, but is characterized by a locally aggressive and infiltrating growth pattern and a high propensity toward local recurrence if incompletely excised.

**Case presentation:**

We report a patient with three post-surgical recurrences of fibromatosis of the breast over a seven year period. The fibromatosis was found to be involving the chest wall musculature and causing persistent and worsening pain. An aggressive operative strategy was undertaken, consisting of mastectomy with en bloc resection of the underlying chest wall musculature, ribs, and parietal pleura.

**Conclusion:**

Aggressive surgical management of fibromatosis of the breast with suspected chest wall involvement is appropriate to attempt to obtain a long-term durable cure.

## Background

Fibromatosis (also synonymous with the term desmoid tumor) is a rare soft tissue tumor that is composed of a bland-appearing proliferation of spindle cells [1,2]. Although fibromatosis is thought to be a benign entity that lacks metastatic potential, it can be characterized by a locally aggressive and infiltrating growth pattern with a high propensity toward local recurrence [3,4]. Fibromatosis arising from within the breast itself is a rare entity. Over the years, there have been a variety of published case reports [5-23]. In addition, there are a few more comprehensive published series describing this entity within the breast [4,24-26]. There seems to be generalized agreement that complete wide excision of fibromatosis that is involving the breast alone is the treatment of choice. However, despite what is reported in the literature, there remains a significant lack of agreement amongst surgeons on how to manage fibromatosis of the breast that is suspected to have concomitant involvement of adjacent chest wall structures. This case report specifically describes the clinical, radiographic, and pathologic features of a patient who experienced three post-surgical recurrences of fibromatosis of the breast over a seven-year period of time secondary to previous inadequate excisions. The fibromatosis was found to be involving the chest wall musculature and causing persistent and worsening pain. An aggressive surgical management strategy was successfully undertaken.

## Case presentation

A 39 year-old white female presented to The Arthur G. James Cancer Hospital with worsening pain of the left breast and left chest wall region and a recurrent palpable mass within the inferior aspect of her left breast. She reports having had three separate left breast biopsies in the past (seven years, four years, and one year prior to her current presentation) for a recurring left breast palpable mass in this same location.

Seven years prior to her current presentation, she presented to an outside community hospital with a palpable left breast mass in the inferior lateral aspect of her left breast. She underwent a left breast biopsy at that time that was reported as showing dense fibrous stroma with fibrocystic changes.

Three years later (four years prior to her current presentation), she noticed a recurrent enlarging palpable left breast mass. She underwent a repeat left breast biopsy by the same surgeon and this showed hyperplastic fibrosis, consistent with fibromatosis of the breast. The pathology report clearly stated that the tumor involved the surgical margins. The patient reports that the surgeon told her that this was a benign tumor and that nothing further needed to be done.

Three additional years later (one year prior to her current presentation), she again noticed a recurrent enlarging palpable left breast mass. She again underwent a repeat left breast biopsy by the same surgeon and this again showed findings consistent with fibromatosis of the breast. Again, the pathology report clearly stated that the tumor involved the surgical margins. The patient reports that the surgeon again told her that this was a benign tumor and that nothing further needed to be done.

Since the time of her last left breast biopsy (one year prior to her current presentation), the patient reports persistent and worsening pain and palpable tenderness within the inferior aspect of her left breast and left chest wall region, with an associated increasing sized palpable mass within the same region.

**Figure 1:**
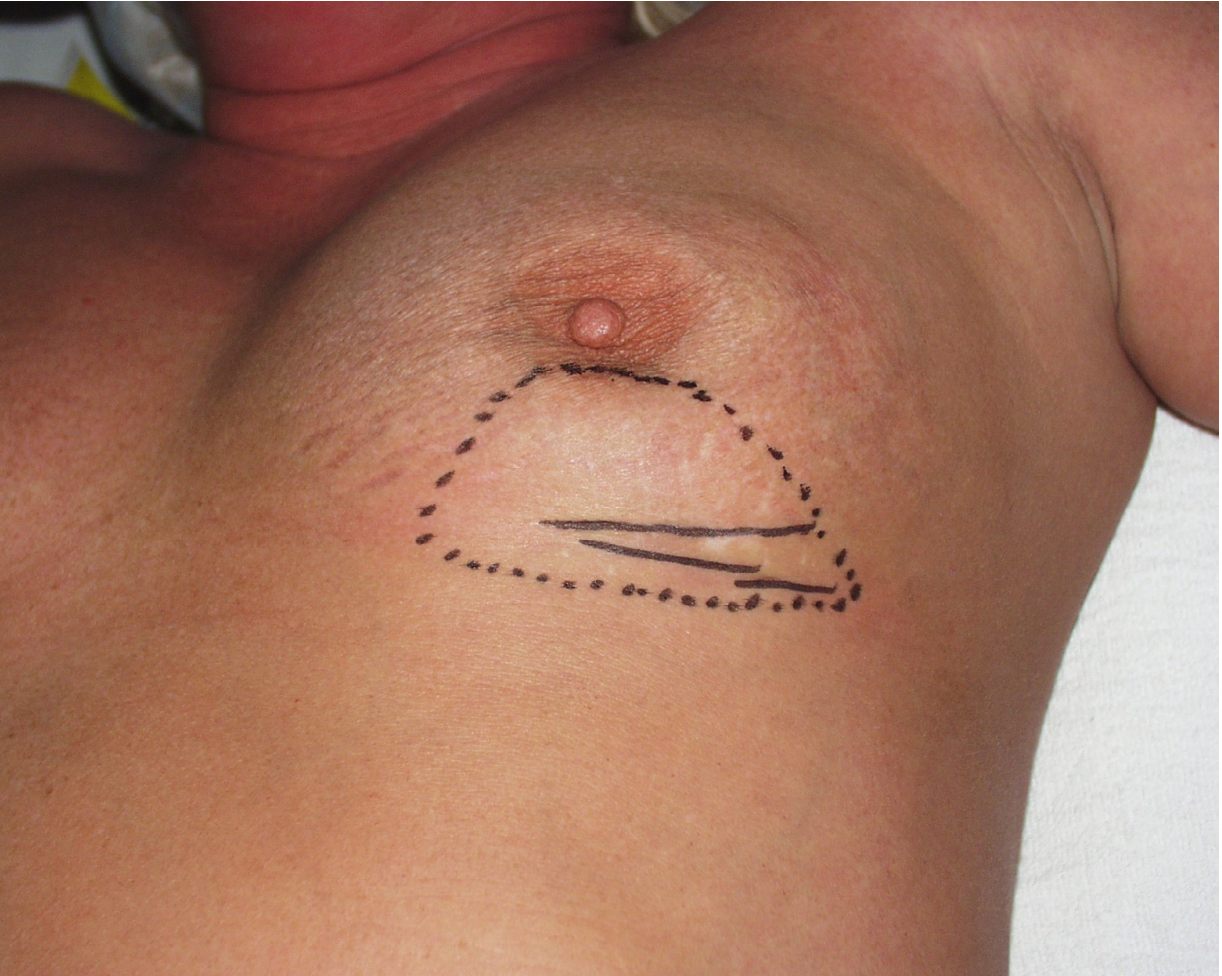
Anatomical location of palpable mass in the left breast (dotted line) and three surgical scars from previous attempted excisions (solid lines).

Upon presentation to The Arthur G. James Cancer Hospital, she was found on clinical examination to have volume loss along the entire inferior aspect of her left breast and slight downward tilting of her left nipple and areolar complex. She had three separate well-healed surgical scars along the inferior-lateral aspect of her left inframammary fold (Figure 1). Underneath these scars, she had a firm palpable mass, clinically measuring 6.5 × 3.0 × 2.5 cm in size and which clinically appeared to be adherent to the underlying left chest wall structures. She had no clinically apparent adenopathy in her left axilla.

**Figure 2:**
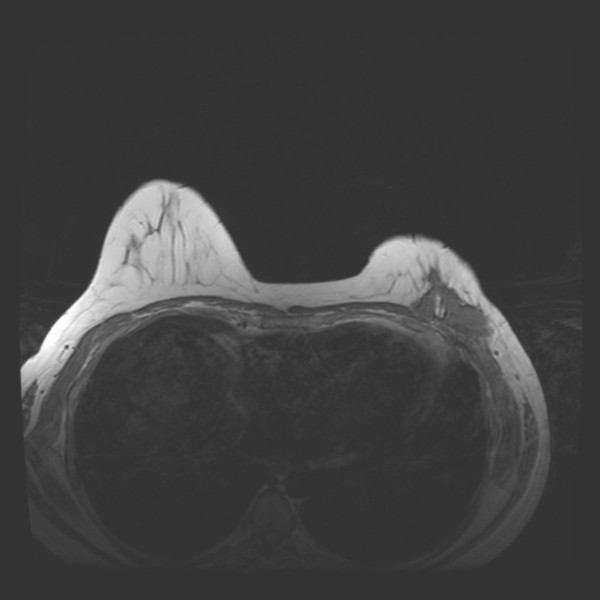
Magnetic resonance imaging non-contrast enhanced T-1 axial view.

**Figure 3:**
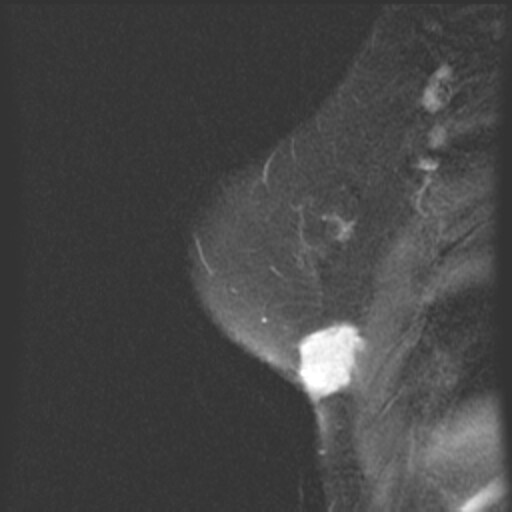
Magnetic resonance imaging contrast enhanced T-1 fat suppression sagittal view.

**Figure 4:**
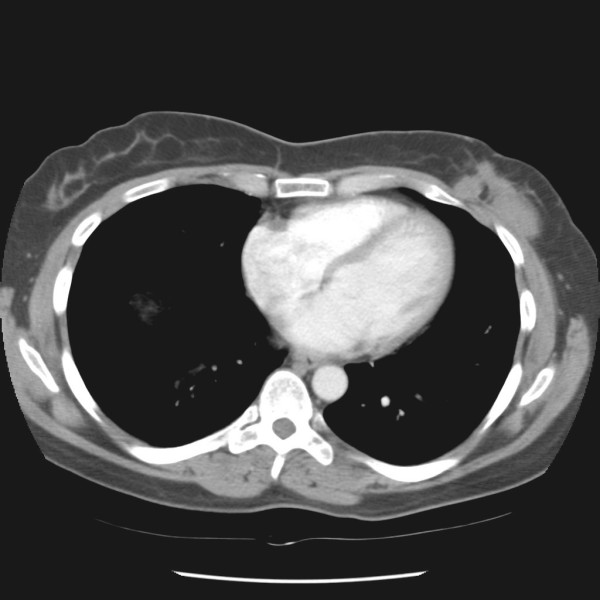
Computed tomography scan axial view.

A mammogram showed scarring and tissue disorganization in the inferior left breast from prior multiple biopsies, but appeared unchanged since a prior mammogram done 13 months previously at an outside community hospital. Magnetic resonance imaging of the left breast showed an intensely enhancing lesion in the inferior-lateral aspect of the left breast, measuring 5.0 × 1.7 cm in size (Figure 2 and 3). This lesion appeared to abut the underlying chest wall musculature and appeared to efface the underlying fat plane. Computed tomography scan of the chest showed a 5.2 × 1.6 cm mass within the inferior-lateral aspect of the left chest wall that appeared to be in continuity with the left pectoralis major muscle and left serratus anterior muscle (Figure 4). A core biopsy was performed to the palpable left breast mass that confirmed the diagnosis of fibromatosis.

**Figure 5:**
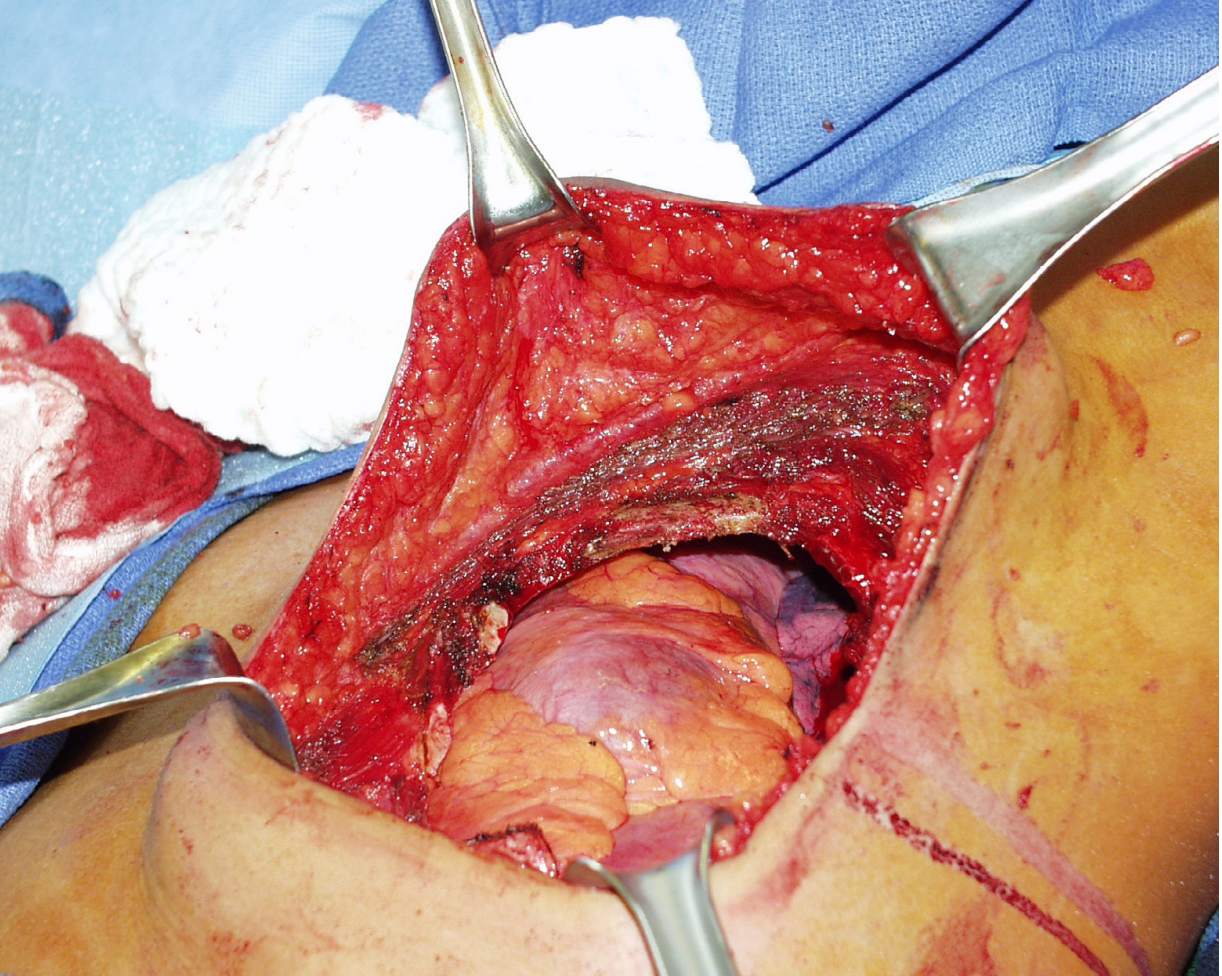
Left chest wall defect created by the en bloc resection, with the patient's head directed towards the upper right corner and the patient's feet directed towards the lower left corner. In the upper right one-quarter of left chest wall defect, deflated left lung is seen. In the lower three-quarters of the left chest wall defect, pericardium and pericardial fat are seen.

The patient was taken to the operating room at to The Arthur G. James Cancer Hospital and underwent a left total mastectomy, with en bloc resection of the underlying musculature (inferior lateral portion of the left pectoralis major muscle, superior portion of the left abdominal oblique musculature, and anterior portion of the left serratus anterior muscle) and en bloc resection of the underlying chest wall structures (fourth, fifth, and six ribs, intercostals muscles, and parietal pleura). The left chest wall defect (Figure 5) was then closed with a 2-mm DualMesh Gore-Tex patch (W. L. Gore & Associates, Inc., Flagstaff, Arizona). The remaining portions of the left pectoralis major muscle was dissected off the underlying left chest wall and its lateral most attachments to the left humerus and superior attachments to the clavicle were divided, allowing it to rotate inferiorly to completely cover the Gore-Tex patch. The left mastectomy site was then closed in the standard fashion. No attempts at cosmetic breast reconstruction with autologous tissue transfer or expander/implant placement were considered at that time. The patient's post-operative course was uneventful and she was discharged to home on post-operative day eight.

**Figure 6:**
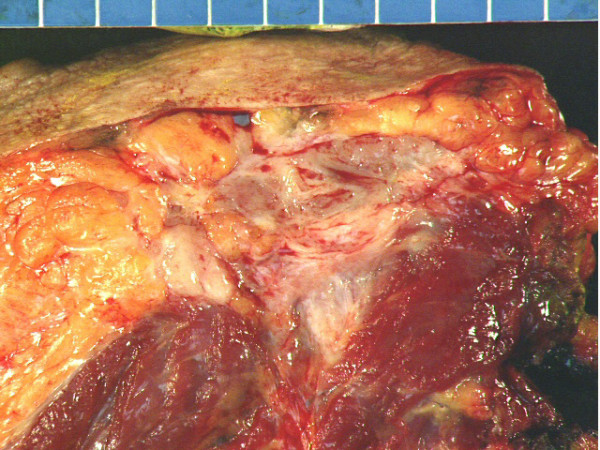
Gross cross-sectional view of pathology specimen.

**Figure 7:**
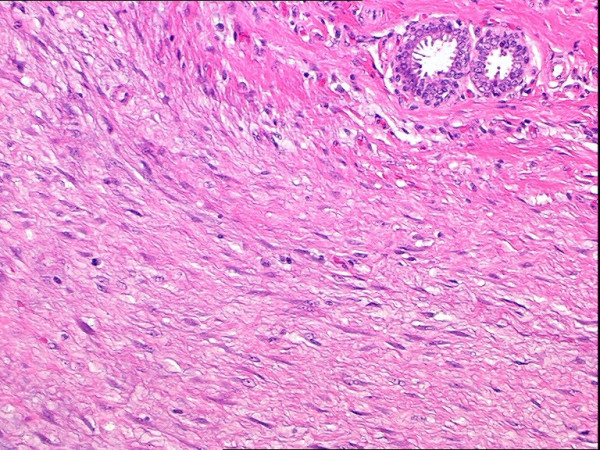
High power (200×) H&E view showing fibromatosis with adjacent mammary ductal epithelium.

**Figure 8:**
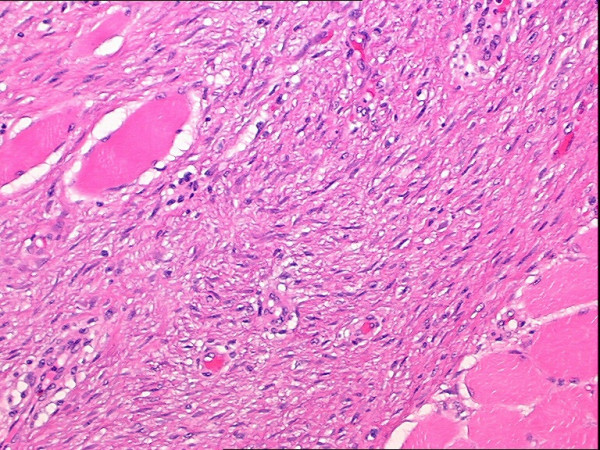
High power (200×) H&E view showing fibromatosis invading into adjacent skeletal muscle.

Gross pathologic evaluation of the specimen, which overall measured 14.5 × 13.3 × 6.4 cm in size, revealed a 5.2 cm tumor that was grossly invading the underlying attached skeletal muscle to a depth of about 1.2 cm (Figure 6). It could not be definitively determined whether the invasion of the underlying skeletal muscle involved only the superficial muscles resected (consisting of the inferior lateral portion of the left pectoralis major muscle, superior portion of the left abdominal oblique musculature, and anterior portion of the left serratus anterior muscle) or whether skeletal muscle invasion was to the level of the underlying intercostal muscles resected. However, both grossly and microscopically, there was no evidence of invasion into the bony ribs or underlying parietal pleura. Microscopic evaluation revealed a proliferation of relatively evenly spaced plump spindle cells arranged in intersecting fascicles and associated with mild to moderate amounts of collagen and occasional mitotic figures (Figure 7) and demonstrated that the spindle cell proliferations invaded into the adjacent skeletal muscle (Figure 8). All surgical margins were negative. On immunohistochemical staining, the spindle cells were negative for S-100 protein, muscle actin (HHF-35), and cytokeratin AE1/AE3. On immunohistochemical staining, less than 5% of the spindle cells were positive for Mib1 (Ki-67). The histology and immunohistochemical staining supported a diagnosis of fibromatosis (desmoid tumor).

The patient is now 22 months out from her previous aggressive operative management of her previous multiple recurrences of her fibromatosis of her left breast and she remains disease free.

## Discussion

In this patient with three post-surgical recurrences of fibromatosis of the breast over a seven year period that were previously inadequately excised and was involving the chest wall musculature and causing persistent and worsening pain, an aggressive surgical resection strategy was undertaken in order to attempt to obtain a long-term durable cure. This aggressive surgical approach has been strongly advocated by some authors for fibromatosis of the breast that involves the chest wall or fibromatosis primarily involving the chest wall [11,27,28]. The factors that support this aggressive surgical approach in appropriately selected patients include the potential of fibromatosis to display a locally aggressive and infiltrating growth pattern into surrounding structures, the resultant high propensity for local recurrence when incompletely excised with positive surgical margins, and the lack of convincing evidence for a proven beneficial role of radiation therapy, chemotherapy, or antiestrogen therapy after incomplete excision of breast fibromatosis. However, other authors [16,19,23,24] have been less enthusiastic and have given only more guarded support for an aggressive surgical approach in appropriately selected patients. The factors that these author stress with regards to their hesitation with proceeding with an aggressive surgical approach are the increased potential for a less than optimal cosmetic outcome, the risks for loss of function, the reported lack of any metastatic potential of fibromatosis, rare reported instances of spontaneous regression of fibromatosis, and reports of recurrent fibromatosis after apparent complete excision with negative surgical margins. However, all authors tend to agree that cosmetic breast reconstruction with autologous tissue transfer or expander/implant placement should not be considered at the initial time of aggressive surgical management and should be delayed for several years while close serial surveillance of the resection bed can be maintained to monitor for evidence of recurrent fibromatosis.

Clearly, early recognition and appropriate complete wide local excision of fibromatosis that is confined to the breast and is without concomitant involvement of adjacent chest wall structures is widely advocated in the literature and is fully embraced by the present authors. This local surgical management strategy will prove to be curative in most cases of fibromatosis involving the breast alone, and can avoid the need for more radical resections to obtain clearance of the surgical margins. However, as is apparent from our reported patient who had preoperative suspicion of involvement of the chest wall musculature, the potential of fibromatosis to display a locally aggressive and infiltrating growth pattern and the resultant high propensity for local recurrence when incompletely excised clearly supports the use of a more aggressive resection approach to assure clearance of the surgical margins and should be considered in similarly selected patients.

## Competing interests

The author(s) declare that they have no competing interests.

## Authors' contributions

**SPP **was the operating surgical oncologist. He was the principle investigator who prepared, organized, wrote, and edited all aspects of the manuscript. **WLM **performed the gross and microscopic pathologic evaluation of the pathology specimen. He prepared all of the histology figures in the manuscript. He read, edited, and approved the final version of the manuscript. **DGS **performed the radiographic evaluation of the patient. He prepared all of the radiographic figures in the manuscript. He read, edited, and approved the final version of the manuscript. **AEA **was the operating thoracic oncologist. He read, edited, and approved the final version of the manuscript. **BAB **was the operating plastic surgeon. He read, edited, and approved the final version of the manuscript. All authors read and approved the final manuscript
